# Effect of different litter materials on some behavioral patterns, growth performance, welfare indices, and carcass traits of Muscovy ducks

**DOI:** 10.1186/s12917-025-04656-5

**Published:** 2025-04-02

**Authors:** Eman Hefnawy, Ahmed Sabek, Eman Elgazzar, Saeed El-Laithy, Souad Ahmed

**Affiliations:** Department of Hygiene and Veterinary Management, Faculty of Veterinary medicine, Benha University, Moshtohour, Benha city, 13736, 13518 Egypt

**Keywords:** Muscovy ducks, Litter type, Behavior, Welfare, Litter microbial load

## Abstract

**Background:**

The current study was conducted to evaluate the effect of different litter materials on growth performance, some behavioral patterns, welfare indicators, and carcass traits in Muscovy ducks. A total of 84 healthy 2weeks old Muscovy ducklings were randomly allocated to 4 groups (3 replicates/ group; each replicate contains 7 birds) according to different litter materials. The first group was reared on wood shavings; the second was reared on sand; the third was reared on chopped rice straw; and the fourth one was reared on wheat straw. Growth performance parameters such as final body weight, body weight gain, feed intake, and feed conversion ratio were evaluated. Some behavioral patterns were recorded when the ducks were 3 weeks old using focal observation 3 days a week, twice per day. Foot pad dermatitis, feather condition score, hock burn, gait score, nostril cleanliness, and carcass traits were evaluated. Litter moisture content, water holding capacity, and microbiological characteristics of different litters were measured.

**Results:**

The results showed that all growth performance parameters of Muscovy ducks were not significantly affected by different litter types (*P* > 0.05). Feeding and leg /wing stretch frequences were significantly higher in ducks reared on wood shavings and sand than other treatments. High pecking and low feather condition score were recorded in birds reared on sand more than other birds. The chopped rice straw group showed the lowest foot pad, gait, and hock burn scores. A little effect of litter types on carcass traits was recorded as only thymus and abdominal fat weights were influenced by different litter types (*P* ≤ 0.05). Sand litter had the lowest water holding capacity, moisture content, total bacterial and fungal counts when compared to other litter types.

**Conclusions:**

In the Muscovy ducks’ sector, alternative bedding materials such as sand, wheat straw, and chopped rice can be used. Sand is the most hygienic litter to be used, as it has the lowest microbial load.

## Background

Duck production is a component of the poultry sector, which is very famous in different parts of the world. Ducks account for the second-largest poultry output in Africa after fowl. In Egypt, there are many pure local duck breeds and other foreign breeds that are used for egg and meat production. The predominant species raised for duck farming is the Muscovy duck which is also a valuable resource for rural populations in developing African countries such as Egypt.

Under deep-litter floor systems, indoor housing requires the provision of bedding materials which may have an impact on productive indices such as growth performance, meat quality, the welfare and well-being of the birds [1].

An essential part of the production process that has a direct impact on the ducklings is the management of the litter as poor quality litter may cause injuries and disabilities among ducks [2]. The bedding material protects the birds from the coldness of the floor, absorbs moisture from drinkers and feces, dilutes fecal chemicals, and ultimately lessens the amount of manure that the birds are exposed to by maintaining the bedding material’s top layer is dry [3].

A good litter must be easily accessible, non-toxic, light weight, absorbent, and inexpensive [4]. The most often utilized litter materials in poultry farms across most nations, including Egypt are wood shavings and wheat straw. These materials’ availability will keep decreasing due to the explosive growth in production of poultry, depletion of natural resources, rivalry with other sectors of the economy, growth in the production of lignocellulosic-based biofuel, progressive ban of the cage system, and usage in animal feed [5, 6], hence, the need to investigate non-traditional litter materials as an alternative to wood shavings and wheat straw is growing.

Numerous materials as peanut shells, rice husks, rice straw, sand, gypsum, shredded and processed paper, corn stalks, coco peat, dried leaves, peat moss, and sand can be used as litter materials in poultry farms [7–12]. Particle size, moisture buildup and content, caking rate, and other physical properties are the most important factors that determine the efficacy of the litter material on growth performance and behaviors of broilers [13]. Litter quality is crucial for respiratory infections, leg and skin health issues, and broiler behaviors and welfare development, low-quality litter has a negative impact on the health, growth performance, and welfare of the birds as well as project revenues, particularly in broiler farms [14, 15].

In broilers, wood shavings and sand improved the welfare, behavior, economic efficiency and growth traits when compared to wheat straw [16]. Using of alternative litter as pumice enhanced the final body weight of broilers with low moisture % [17]. At 42 days old, the foot pad dermatitis decreased in chickens reared on wood shaving + acidic pumice stone than those reared on acidic pumice alone [17].

Ducks reared on different litter materials displayed various responses in their growth performance and carcass parameters [12]. Low litter quality may lead to hock, foot, and breast lesions. In Europe, foot, hock, and breast burn lesions are frequently used in animal welfare audits as a gauge for the general well-being of the birds and their housing [18]. Few studies have examined the impact of different litter materials on Pekin and Mullard ducks. In Mullard ducks, aggressive behavior was higher in ducks reared on sand than those reared on wood shaving, also the type of litter (wood shaving and sand) had no significant effect on final body weight, feed intake, nostril cleanness, feather score, foot pad, and gait of ducks [11]. In Pekin ducks final body weight was higher in birds reared on rice husks than those reared on saw dust and cocopeat respectively, unlike carcass % that was not significantly affected by litter [12].

In general, In Muscovy ducks the reports about the effect of different managemental factors on behavior and welfare are rare so we conducted a bundle of studies that started with our previously published study [19] in which we evaluated the effect of different light colors on Muscovy ducks. There is not any report about the influence of litter materials on Muscovy ducks. Therefore, this study was conducted to evaluate the effect of different litter materials (wood shaving, sand, chopped rice straw, and wheat straw) on growth performance, behavior, welfare, and carcass quality of growing Muscovy ducklings.

## Materials and methods

The study was conducted at Faculty of Veterinary medicine, Benha University, Egypt. The study was carried out according to the guidelines for the care and use of animals. The study protocol was approved by the Scientific Ethics Committee of Faculty of Veterinary medicine, Benha University, Egypt (BUFVTM 27-09-23).

### Birds and management

A total of 84 healthy Muscovy ducklings aged 2 weeks with an average body weight of 302.83 ± 2.83 g were purchased from a private local company in Egypt. Birds were housed in previously cleaned and disinfected four symmetrical pens; each pen’s dimensions were 3.75 m length, 3.6 m width, and 3 m height which was divided into 3 parts one for each replicate using wooden barriers with a total space area of 4.5 m^2^ for each replicate (each bird had a floor space about 0.6 m^2^). During the experimental phase, the average room temperature was 29.33 ± 0.11 °C, the relative humidity was 50.10 ± 0.46%, and the photoperiod was 16 h light and 8 h dark. All ducklings were vaccinated against avian influenza and fowl cholera at the age of 4 and 6 weeks, respectively. Feeders and drinkers were equally distributed in the pens, and clean, fresh water was available throughout the day. From 2 to 4 weeks old, a starter diet contained 22% crude protein was given [11] followed by a grower diet contained 19% crude protein as recommended by [20] from 5 to 10 weeks old, the food was offered 2 times per day ad libitum.

### Experimental design

A total of 84 healthy Muscovy ducklings aged 2 weeks were randomly allocated to 4 groups according to different litter materials; each group contained 21 birds divided into 3 replicates (7 birds each). Each group was housed in a separate pen with dimensions were 3.75 m length, 3.6 m width, and 3 m height which was divided into 3 parts one for each replicate using wooden barriers with a total space area of 4.5 m^2^ for each replicate. The birds in the first group were reared on wood shavings; the birds in the second group were reared on sand; the birds in the third group were reared on chopped rice straw; and the birds in the fourth group were reared on wheat straw. The thickness of the litter in each group was 10 cm. The study was conducted during the growing period of Muscovy ducklings, from 2 to 10 weeks old.

### Growth performance parameters

Five birds from each replicate (15/ group) were chosen at random at the end of the trial, and their final body weights were determined (FBW) in grams using digital balance. Total feed intake in grams was determined by deducting the leftover quantity from the weekly amount fed to each group of birds. Individual feed intake was calculated by dividing the total amount of food consumed / total number of birds per group.

Body weight gain (BWG) was calculated by deducting the initial body weight from the final body weight. FCR of 15 birds that their body weight gains were measured was calculated by dividing the feed intake / body weight gain.

### Behavioral observation

Ducklings were given 1 week for adaptation, so behavioral observation started when the ducks were 3 weeks old. Fifteen birds from each group (5 per replicate) were randomly selected and marked by leather leg bands for behavioral observation. The behavioral patterns of each group were recorded 3 days a week, twice per day, at 9.00–10.00 am and 2.00–3.00 pm. Each bird’s behavioral patterns were observed by focal observation for 3 min, with a total observation time of 15 min per replicate per group in the morning and in the afternoon. The behavioral descriptions are displayed in Table 1.

**Table 1:** Descriptions of behavioral patterns of ducks

Behavior	Description
**Feeding**	The bird inserts its bill into the feeder to consume feed [42].
**Drinking**	The bird inserts its bill into the drinker to consume water [42].
**Standing**	The legs are in contact with the floor without any activity [11].
**Sitting**	Ducks lie on the ground with open or closed eyes [11]
**Walking**	The bird moves from one point to another without contributing to other activities [43].
**Litter scratching**	The bird scratches the floor by its leg [11].
**Object pecking**	Ducks peck the ground or other parts of the pen by their beak [43].
**Head shaking**	Complete lateral movement of the head [43].
**Preening**	The bird cleans its plumage using the beak [43].
**Wing and leg stretching**	The bird stretches the wing and the leg of the same side [43].
**Tail wagging**	The tail moves from side to side [43].
**Feather pecking**	The bird pecks, pulls or sometimes eats the feather of other individual [42].

### Foot pad quality and feather condition score

To follow up the influence of different litter types on foot pad and feather condition score, 15 birds per group (5 / replicates) were randomly selected, the foot pad quality and feather condition scores of these birds were determined at 3, 6, and 10 weeks old using a score scale as follows: Foot pad: score 0 represents the best condition, with no lesions or embedded dirt on the heel or toe pads; score 1 represents a moderate condition, with callused or cracked pads that have lesions covering less than 50% of the pad area and no blood; score 2: worst: any bleeding lesions or calluses that cover 50% or more of the pads. Feather condition: Score 0: good, indicates full feathering; score 1: moderate, indicates slight feather pecking, slight damaged areas less than 1 cm2; score 2: bad, indicates severe feather pecking, bleeding, and severe damaged areas more than 2 cm^2^ [2].

### Hock health, gait score, and nostril cleanliness

The hock burns of 15 birds per group (5/replicate) were assessed at the last week of the trial when the ducks were between 9 and 10 weeks old using the following score system [21]; scores: 0, good, unaffected hock; 1, slight discoloration or lesions; 2, severe scabbing and lesions. The gait score of 15 birds per group (5 replicates) was calculated at the last week of the trial when the ducks were between 9 and 10 weeks old using the following score scale: score 0: duck walks and waddles the best; score 1: mild; ducks walk with a slight limp or have a laborious gait as a result of crossed feet or bowlegs; score 2: severe; ducks are hesitant to walk and will only go short distances when prodded, usually as a result of evident leg issues (synovitis, severely crossed feet, or very bending the legs).

The nasal cleanliness of 15 birds per group (5 repetitions) was assessed at the last week of the trial when the ducks were between 9 and 10 weeks old using the following score scale: The lowest score is 1, which indicates that the nostrils are obstructed by mucus or dust; the highest score is 0, which indicates that the nostrils are clear and clean [2].

### Carcass traits

At the end of the trial 7 male birds were randomly selected from each group and their live body weights were determined. Ducks were held with their heads down and their wings and legs restrained to prevent vigorous movement. Slaughtering was done using a sharp knife that made a single cut across the neck, cutting the carotid arteries, jugular veins, esophagus, trachea, and the connective tissues of the neck. Knife sharpness is very important during slaughtering birds without pre- slaughter anesthesia or stunning as it promotes better bleeding and reduces discomfort and anxiety in birds by inducing rapid unconsciousness as recommended by [19]. Measurements were made for the hot carcass. Carcass weight was recorded in grams using digital balance. Dressing % equals carcass weight / live body weight × 100. The weights of the immune organs (spleen, thymus, and bursa of Fabricius), as well as the giblets’ (liver, heart, and gizzard, and abdominal fat) in grams using a digital balance.

### Litter quality traits

#### Litter sampling

Throughout the trial, a composite sample of litter was taken every two weeks from each of the four distinct litter groups. The litter samples were collected from five different sites within each pen. Each pen was placed in five different spots, including the center and the four corners, according to [22].

#### Water holding capacity

It was calculated at zero day by weighing each litter and putting it in a container, adding water and letting it sit for half an hour. After draining the excess water, the samples were weighed to calculate the percentage of water absorbed on a dry matter basis [23].

#### Litter moisture content

It was determined every 2 weeks as follows: five grams of each litter sample were dried in a drying oven for 48 h at 60 °C for partial dryness, and for 5 h at 105 °C for complete dryness. The samples were cooled and weighed in accordance with [24].

### Litter microbiology

#### The total bacterial count

The following was done in accordance with the pour-plate approach as stated by [25]: 1 g of each litter treatment was taken and put in a sterile tube with 9 ml of sterile physiological saline. Aseptic preparation of 10-fold serial dilutions was then carried out. A sterile Petri plate was aseptically filled with 1 ml from each of the 2 dilutions 10^− 6^ and 10^− 8^. Each Petri dish was aseptically filled with 10 ml of melted plate count agar that had been chilled to 45–50 °C and thoroughly mixed horizontally. The plates were incubated at 37 °C for 24 h following solidification.

#### The total fungal count

1 g was taken from each litter treatment and placed in a sterile tube containing 9 ml sterile physiological saline. Aseptic preparation of 10-fold serial dilutions was then carried out. 1 ml from the previously prepared 10-fold dilutions was put onto a sterile Petri dish. Each dish was aseptically filled with 10 ml of sterile Sabouraud’s dextrose agar at 40 °C. The inoculation plates were thoroughly mixed, allowed to harden, and then darkly incubated at 25 °C for 3 to 5 days in accordance with [26], the plates were inspected to determine the fungal count.

### Statistical analysis

SPSS version 22 was used to analyze the collected data. Growth performance, Behavioral patterns, welfare parameters, carcass traits, and litter characters were analyzed using analysis of variance (ANOVA). The normality of the data distribution was evaluated by a Shapiro-Wilk test. Means and standard error means were used to present the data. *P* ≤ 0.05 was used to declare the data to be different.

## Results

### Growth performance

As shown in Table 2 the different litter materials had no significant effect on duck growth performance parameters including FBW, feed intake, BWG, and FCR (*P* > 0.05). However, ducks reared on chopped rice straw displayed numerically higher FBW, BWG, and FCR than other treatments.

**Table 2:** Effect of different litter materials on growth performance of Muscovy ducks in growing period

Growth performance	Litter materials				
	Wood shavings	Sand	Chopped rice straw	Wheat straw	*P* - value
Initial BW (2^nd^ week) (g)	300.33 ± 7.92	304.67 ± 7.92	304.33 ± 7.92	302.00 ± 7.92	0.97
Final BW (10^th^ week) (g)	3063.00 ± 183.61	3004.00 ± 183.61	3212.00 ± 183.61	3198.00 ± 183.61	0.81
BWG (g)	2762.67 ± 60.37	2699.33 ± 60.37	2907.67 ± 60.37	2896 ± 60.37	0.08
Feed intake (g)	2945.61 ± 321.04	2866.8 ± 321.04	2831.95 ± 321.04	3081.59 ± 321.04	0.06
FCR	1.06 ± 0.57	1.06 ± 0.57	0.97 ± 0.57	1.06 ± 0.57	0.5

Least square means (± SE) with different superscripts letters in the same row are significantly different at *p* ≤ 0.05. BW: body weight; BWG: body weight gain; FCR: feed conversion rate

### Behavioral patterns

The litter materials affected significantly on some behavioral patterns of ducks such as feeding, sitting, walking, standing, leg / wing stretch, feather pecking, and object pecking. While there was no significant effect of them on drinking, preening, head shaking, tail wagging, and litter scratching. The highest frequencies of feeding, standing, walking, and feather pecking were observed on ducks reared on sand. Ducks reared on wood shavings and sand displayed the highest frequency of leg and wing stretch than those reared on other litter types (*P* = 0.004). The object pecking was significantly affected by litter types as ducks reared on chopped rice straw displayed the lowest object pecking when compared to those reared on sand, wheat straw, and wood shavings respectively (*P* = 0.005) (Table 3).

**Table 3:** Effect of different litter materials on some of behavioral patterns of Muscovy ducks in growing period

Behavioral patterns frequency	Litter materials				
	Wood shavings	Sand	Chopped rice straw	Wheat straw	*P* - value
Feeding	0.42^ab^ ± 0.06	0.57^a^ ± 0.06	0.34^b^ ± 0.06	0.38^b^ ± 0.06	0.03
Drinking	0.64 ± 0.07	0.63 ± 0.07	0.61 ± 0.07	0.48 ± 0.07	0.39
Sitting	2.02 ^a^ ±0.07	1.80^b^ ± 0.07	1.81^b^ ± 0.07	1.99^ab^ ± 0.07	0.03
Standing	0.71^b^ ± 0.08	1.32^a^ ± 0.08	0.81^b^ ± 0.08	0.79^b^ ± 0.08	< 0.001
Locomotion	0.61^b^ ± 0.07	1.13^a^ ± 0.07	0.61^b^ ± 0.07	0.54^b^ ± 0.07	< 0.001
Preening	1.06 ± 0.09	0.94 ± 0.09	1.07 ± 0.09	1.12 ± 0.09	0.57
Wing & Leg stretch	0.54^a^ ± 0.05	0.42^ab^ ± 0.05	0.32^b^ ± 0.05	0.31^b^ ± 0.05	0.004
Head shaking	0.41 ± 0.05	0.40 ± 0.05	0.41 ± 0.05	0.38 ± 0.05	0.97
Tail wagging	0.60 ± 0.06	0.60 ± 0.06	0.63 ± 0.06	0.59 ± 0.06	0.96
Feather pecking	0.22^b^ ± 0.06	0.50^a^ ± 0.06	0.05^c^ ± 0.06	0.09^bc^ ± 0.06	< 0.001
Litter scratching	0.38 ± 0.05	0.24 ± 0.05	0.43 ± 0.05	0.30 ± 0.05	0.07
Object pecking	0.31^a^ ± 0.04	0.21^ab^ ± 0.04	0.11^b^ ± 0.04	0.24^a^ ± 0.04	0.005

Least square means (± SE) with different superscripts letters in the same row are significantly different at *p* ≤ 0.05

### Foot pad quality and feather condition score

As shown in Table 4, the significant effect of litter types on foot pad quality started at the age of 6 weeks till the end of the trial as ducks reared on chopped rice straw displayed inferior foot pad quality when compared to other treatments. Unlike feather condition score that lower in ducks reared on sand than those reared on other litter materials.

**Table 4:** Effect of different litter materials on foot pad quality and feather condition score of Muscovy ducks in growing period

	Litter materials				
	Wood shavings	Sand	Chopped rice straw	Wheat straw	*P* - Value
Foot pad quality					
3^rd^ week	0.00	0.00	0.00	0.00	0.10
6^th^ week	0.00^b^	0.00^b^	1.00^a^	0.00^b^	0.001
10^th^ week	1.00^b^	1.00^b^	2.00^a^	1.00^b^	0.03
Feather condition score					
3^rd^ week	0.00	0.00	0.00	0.00	0.10
6^th^ week	0.00^b^	1.00^a^	0.00^b^	0.00^b^	0.004
10^th^ week	0.00^b^	1.00^a^	0.00^b^	0.00^b^	0.008

Scores with different superscripts letters in the same row are significantly different at *p* ≤ 0.05

### Hock burn, gait score, and nostril cleanliness

Hock burns were significantly affected by litter type (*P* = 0.001). Ducks reared on wood shavings and sand showed better hock condition than those reared on chopped rice straw and wheat straw. Ducks reared on wood shavings and wheat straw walked freely without detection of any issues, while ducks reared on sand and chopped rice straw displayed an abnormal gait. Nostril cleanliness was not affected by the different litter materials (*P* > 0.05) (Table 5).

**Table 5:** Effect of different litter materials on Hock burns score, gait score and nostrils cleanliness of Muscovy ducks in the last week of growing period

Item	Litter materials				
	Wood shavings	Sand	Chopped rice straw	Wheat straw	*P* - value
Hock burns	0.00^b^	0.00^b^	1.00^a^	1.00^a^	0.001
Gait score	0.00^b^	1.00^a^	1.00^a^	0.00^b^	0.03
Nostril cleanliness	1.00	1.00	1.00	1.00	0.100

Scores with different superscripts letters in the same row are significantly different at *p* ≤ 0.05

#### Carcass traits

Live body weight, carcass weight, dressing %, heart weight, liver weight, gizzard weight, spleen weight, and bursa of fabricious weight of ducks were not significantly affected by different litter materials. ducks. Litter materials showed a significant effect on thymus weight; The highest thymus weight was observed on ducks reared on whrat straw followed with those reared on sand, then those reared on chooped rice straw and the lowest weight was observed in ducks reared on wood shavings (*P* = 0.03). Abdominal fat weight was significantly affected by litter materials; it was higher in birds reared on sand, wood shavings, and wheat straw than those reared on chopped rice straw (*P* = 0.05) (Table 6).

**Table 6:** Effect of different litter materials on carcass parameters of Muscovy ducks at the end of growing period

Carcass parameters	Litter materials				
	Wood shavings	Sand	Chopped rice straw	Wheat straw	*P*– Value
Live body weight (g)	3189.00 ± 268.77	3303.00 ± 268.77	3179.00 ± 268.77	3400.00 ± 268.77	0.92
Carcass weight (g)	2190.00 ± 190.23	2276.00 ± 190.23	2259.00 ± 190.23	2290.00 ± 190.23	0.98
Dressing %	68.67 ± 1.73	68.90 ± 1.73	71.06 ± 1.73	67.35 ± 1.73	0.63
Heart (g)	17.64 ± 1.37	18.76 ± 1.37	19.38 ± 1.37	20.54 ± 1.37	0.52
Liver (g)	76.76 ± 9.81	87.26 ± 9.81	63.84 ± 9.81	73.30 ± 9.81	0.42
Gizzard (g)	65.63 ± 5.73	81.78 ± 5.73	73.67 ± 5.73	75.28 ± 5.73	0.28
Spleen (g)	3.05 ± 0.40	3.04 ± 0.40	3.08 ± 0.40	3.03 ± 0.40	1.00
Thymus (g)	12.60^b^ ± 1.30	16.85^a^ ± 1.30	15.19^ab^ ± 1.30	18.16^a^ ± 1.30	0.03
Bursa of fabricious (g)	2.33 ± 0.85	2.04 ± 0.85	4.00 ± 0.85	3.76 ± 0.85	0.27
Abdominal fat (g)	32.19^ab^ ± 3.13	35.45^a^ ± 3.13	23.25^b^ ± 3.13	27.40^ab^ ± 3.13	0.05

Least square means (± SE) with different superscripts letters in the same row are significantly different at *p* ≤ 0.05

### Litter characters

#### Moisture content and water holding capacity of different litter materials

Throughout the study, the lowest moisture content was recorded on sand when compared to other litter materials (*P* < 0.001), while the highest content was observed on chopped rice and wheat straw litters. The water holding capacity of sand litter was the lowest among the different litter materials used in this trial (*P* < 0.001) (Table 7).

**Table 7:** Litter moisture content (%) and water holding capacity (%) of different litter materials in growing period of Muscovy ducks

Moisture content (%)	Litter materials				
	Wood shavings	Sand	Chopped rice straw	Wheat straw	*P* - value
2^nd^ week (zero day)	4.67^b^ ± 0.58	1.33^c^ ± 0.58	6.67^a^ ± 0.58	6.00^ab^ ± 0.58	0.001
4^th^ week	21.33 ^b^ ±2.31	4.67 ^c^ ±2.31	34.00 ^a^ ±2.31	31.33^a^ ± 2.31	< 0.001
6^th^ week	46.00^b^ ± 4.42	9.33^c^ ± 4.42	66.67^a^ ± 4.42	52.00^b^ ± 4.42	< 0.001
8^th^ week	50.67^b^ ± 4.87	10.67 ^c^ ±4.87	72.00^a^ ± 4.87	60.67 ^ab^ ±4.87	< 0.001
10^th^ week	62.67^a^ ± 5.55	6.67^b^ ± 5.55^b^	65.33 ^a^ ±5.55	65.33 ^a^ ±5.55	< 0.001
Water holding capacity (%)	260.17 ^a^ ±16.10	23.17^c^ ± 16.10	162.33 ^b^ ±16.10	266.83 ^a^ ±16.10	< 0.001

Least square means (± SE) with different superscripts letters in the same row are significantly different at *p* ≤ 0.05

#### Microbiological characters of different litter materials

The different litter materials significantly affected total bacterial and total fungal counts as documented in Table 8. The highest total bacterial count was recorded in chopped rice straw at 4th and 8th weeks of age. The lowest total bacterial count was recorded in sand litter during the 2nd, 4th, 8th and 10th weeks of age. Total fungal count at the 2nd and 10th weeks of age was not significantly affected by the litter types. On the other hand, from the 4th to the 8th weeks of age, sand litter showed the lowest total fungal count among the litter treatments.

**Table 8:** Total bacterial count and total fungal count of different litter materials in growing period of Muscovy ducks

	Litter materials				
	Wood shavings	Sand	Chopped rice straw	Wheat straw	*P* - value
Total bacterial count ×10^7^ CFU/g					
2^nd^week (zero day)	2.69^a^ ± 0.10	2.26^b^ ± 0.10	2.82^a^ ± 0.10	2.65^a^ ± 0.10	0.02
4^th^ week	7.70 ^b^ ±0.08	7.15 ^c^ ±0.08	8.03 ^a^ ±0.08	7.75 ^b^ ±0.08	< 0.001
6^th^ week	7.69^b^ ± 0.10	7.48^b^ ± 0.10	8.30^a^ ± 0.10	8.05^a^ ± 0.10	0.002
8^th^ week	8.03^c^ ± 0.04	7.83^d^ ± 0.04	8.33^a^ ± 0.04	8.16^b^ ± 0.04	< 0.001
10^th^ week	7.78^b^ ± 0.09	7.13^c^ ± 0.09	8.08^a^ ± 0.09	8.14^a^ ± 0.09	< 0.001
Total Fungal count×10^7^ CFU/g					
2^nd^ week (zero day)	2.26 ± 0.09	2.00 ± 0.09	2.36 ± 0.09	2.20 ± 0.09	0.10
4^th^ week	7.30^a^ ± 0.10	6.68^b^ ± 0.10	7.33^a^ ± 0.10	7.29^a^ ± 0.10	0.004
6^th^ week	7.27^ab^ ± 0.14	6.81^b^ ± 0.14	7.51^a^ ± 0.14	7.38^a^ ± 0.14	0.04
8^th^ week	7.50^a^ ± 0.08	7.18^b^ ± 0.08	7.67^a^ ± 0.08	7.53^a^ ± 0.08	0.01
10^th^ week	7.08 ± 0.13	6.58 ± 0.13	6.88 ± 0.13	7.01 ± 0.13	0.09

Least square means (± SE) with different superscripts letters in the same row are significantly different at *p* ≤ 0.05

## Discussion

### Growth performance

The body weight of ducks was not significantly affected by different litter materials. However, the obtained results numerically revealed that ducks reared on chopped rice straw displayed the highest final body weight when compared to other treatments.

The insignificant difference in body weight between ducks in different groups may attributed to receiving the same ration and the same housing management. In consistent with the obtained results, White Pekin ducks body weight was not significantly affected by bedding types (cocopeat, rice husks, and sawdust) from day one to 35 days [12]. The final body weight of Mullard ducks reared on wood shavings and sand was not significantly different as recorded by [11].

In broilers, the different bedding materials had no effect on live body weight (*P* > 0.05) [27]. Our findings agree with [28] who found no significant effect of bedding materials (wheat straw, clover straw, and corn stalk straw) on the body weight of turkeys of all ages. The same effect of different litter materials on body weight was recorded in quails [29].

In contrast, Abougabal and Taboosha [16] recorded the significant effect of different litter materials on the live body weight of broiler chicks, as at the age of 6 weeks, chicks reared on wood shaving and sand displayed more body weight than those reared on wheat straw. The same significant effect of bedding materials on the body weight of broilers was described by [30].

The current results revealed that the different bedding materials had no significant effect on BWG, feed intake, and or FCR of Muscovy ducks during the growing period. The result agrees with [11] who found no significant effect of wood shaving, plastic slatted, and sand on weekly feed intake and FCR of Mullard ducks. The same results were recorded in broilers [27].

On the other hand, in broilers, using different bedding materials significantly affected body weight gain, feed intake, and feed conversion ratio [16].

### Behavioral patterns

One of this study’s aims is to evaluate the effect of bedding materials on some behavioral patterns of Muscovy ducks. Using sand as bedding material improved feeding in ducks when compared to wood shaving, wheat straw, and chopped rice straw, respectively. This significant difference in feeding frequency may be attributed to the physical characteristics of the litter, which may affect the ration quality and encourage birds to move toward the feeders.

This agrees with [13] who recorded an improvement in feeding in broiler chickens reared on sand compared to those reared on wood shaving. In Mullard ducks, using different litter materials affected feeding frequency significantly [11]. In contrast, different bedding materials had no effect on the feeding of broilers [31].

In the current study, litter materials showed no effect on drinking behavior as this behavior may be affected by other managemental factors than litter. A lot of previous studies revealed the same effect of litter materials on drinking behavior [31, 32]. Our results disagree with the findings of [11, 13] who observed the significant impact of bedding material on drinking frequency of broiler chickens and ducks respectively. Some behaviors are not related with activities as sitting and standing, in the current study sitting frequencies were significantly affected by the type of litter as ducks reared on wood shaving showed the highest sitting bouts followed by those reared on wheat straw, chopped rice straw and sand respectively.

Ducks may prefer to rest on wood shavings due to its soft effect on the feet and plumage of birds, as well as their cleanliness. In consistent to our finding, Ramadan et al. [33] stated that the highest percentage of broiler chicks displayed sitting was observed in wood shaving group compared to other groups (Straw, sand, wood shaving + straw, wood shaving + sand, and straw + sand).

On the contrary, in Mullard ducks there was no difference in sitting behavior between birds reared on wood shaving and birds reared on sand [11]. The highest standing and walking bouts were observed in birds reared on sand when compared to other treatment groups; this may be attributed to the physical characteristics of the litter that encourage birds to stand or walk rather than rest. This result is in agreement with [34, 35] who documented that broiler chickens reared on sand showed more standing and locomotion than those reared on wood shaving.

On the other hand, wood shaving and sand had no effect on the standing of Mullard ducks [11] this may be due to the breed difference between Mullard and Muscovy ducks. Wood shaving and sand had no effect on the locomotion and sanding of broilers [13]. Our results disagree with [33] who found that the percentage of chicks performing standing and walking was higher in straw litter than wood shaving and sand litter.

Preening, head shaking, and tail wagging are comfort behaviors; in the current study these behaviors were not significantly affected by bedding materials; other factors may have an impact on these behaviors than litter. It is in line with the previous studies, which revealed no impact of bedding material on broiler preening [31, 33] or Pekin ducks [36]. In contrast, broilers reared on wood shaving showed more preening than those reared on sand and rice hulls [13]. In Mullard ducks, litter type affected significantly on preening as described by [11].

Wing and leg stretch was significantly affected by litter type. Birds reared on wood shavings displayed more wing and leg stretch than those reared on sand, chopped rice straw, and wheat straw, respectively. Leg and wing stretching in broilers significantly differed between litter treatments [37]. On the other hand, different litter materials had no significant effect on activities like stretching in broilers [13] or Mullard ducks [11]. Stereotypic behaviors like litter scratching and object pecking showed a different response to the litter type, as object pecking was significantly affected by litter while litter scratching was not affected.

Agonistic behavior represented by feather pecking was observed more in ducks reared on sand than in those reared on wood shaving, wheat straw, and chopped rice straw, respectively. Low feather pecking on chopped rice straw and wheat straw groups may be attributed to the high fiber content of these materials, which act as an external and extra source of fiber for ducks.

In consistent with the current findings, Mohammed et al. [11] reported a higher aggression frequency in Mullard ducks reared on sand than those reared on wood shaving. Pecking increased in chickens reared on sand than in those reared on wood shavings [36]. Our result disagrees with previous studies that reported no effect of different litter types on the agonistic behavior of birds [16, 31, 33].

### Foot pad quality, feather condition score, Hock burns, gait score, and nostril cleanliness

Foot pad quality, feather quality, hock burns, gait score, and nostril cleanliness were used to evaluate the effect of different litter materials on Muscovy ducks’ welfare. The foot pad quality was significantly affected by the litter type from the 6th week of age. Birds reared on chopped rice straw showed moderate foot pad quality when compared to other birds reared on other litters that displayed the best foot pad quality. Within the last week of the study, foot pads showed inferior quality in ducks reared on chopped rice straw. It is suggested that chopped rice straw has a high moisture content that causes foot dermatitis.

The findings agree with [38] who stated that foot pad score of broilers reared on wood shaving was better than those reared on straw. Rearing broilers on sand improved their foot pad score as mentioned by [16]. In contrast, bedding had no effect of foot pad of Mullard ducks [11] or broilers [33].

The litter type had a great impact on the feather condition score of ducks at 6 and 10 weeks old, as birds reared on sand showed a moderate score compared to other treatment groups, which showed a good score. This may be attributed to the higher frequency of feather pecking of ducks reared on sand. Contrary to the current study results, in Mullard ducks, there was no effect of litter type on feather quality [11]. Also, broilers reared on sand showed the best feather condition score [16].

Birds reared on wood shavings and sand showed better hock conditions than those reared on chopped rice and wheat straw, as these materials hold water and retain moisture, which increases the incidence of hock burns. This result confirms the low footpad score caused by chopped rice straw. The result agrees with [27] who found no difference in hock burn incidence between broilers reared on wood shavings and sand. In contrast, there was no effect of different litter types on hock burns in turkeys [28] or broilers [33].

Ducks reared on wood shaving and wheat straw displayed a normal gait compared to those reared on sand and chopped rice straw, which showed a mildly abnormal gait. The abnormal gait observed on sand and chopped rice groups may be attributed to the physical characteristics of these litters, which may retire the ducks from normal locomotion. Unlike our study [11, 39], stated that litter had no effect on gait score of Mullard ducks or broiler chickens respectively. Throughout the study, the effect of litter was not significant on nostril cleanliness. In agreement with our study, the nostril cleanliness of Pekin and Mullard ducks was not affected by litter [2, 11].

### Carcass traits

Most carcass traits were not significantly affected by litter, except thymus and abdominal fat weights. Live body weight, carcass weight, dressing percent and giblet weights may mostly be affected by other factors such as the feeding system or light regime. Previous studies conducted on broilers, turkeys, and ducks, demonstrated that different litter materials had no significant effect on carcass weight, or percent, gizzard weight, or bursa of fabricious weight [12, 13, 28].

In contrast, live body weight and carcass weight were higher in broilers reared on wood shaving and sand than those reared on wheat straw [16] also [30], reported higher carcass weight and gizzard yield in broilers reared on wood shaving than birds reared on sand. Unlike to our findings, fat weight of broiler chickens reared on different bedding materials were not significantly affected (*P* > 0.05) [39].

### Litter parameters

The physical and microbiological characteristics of the litter are very important to improve the hygienic conditions of the birds. The moisture content and water holding capacity of the various litters employed in the current investigation varied significantly from one another.

Throughout the course of the investigation, sand displayed the lowest percentages of both moisture content and water holding capacity when compared to other litters. This may be attributed to large particles of sand losing water quickly. The high moisture content and water holding capacity of chopped rice straw, wheat straw, and wood shavings make them good media for microorganisms’ growth.

The lowest total bacterial and fungal count was recorded in sand litter. Farghly et al. [6] recorded the difference in physical parameters of the different litters used in broilers. In the same line with the current findings, sand had lower moisture content than straw and wood shaving [16]. The microbial loads differ from one litter to other [39]. Pine shaving had a higher mold and bacterial counts than sand [40]. In turkey, straw based litters (rice straw and wheat straw) had higher total bacterial and total fungal counts than sand litter [41].

## Conclusion

In conclusion, wood shavings remain the preferred litter for most poultry farms; nevertheless, different litter materials such as sand, chopped rice straw, and wheat straw could be used. Among the different materials used in the current study, sand litter is the best one to used as a substitute to wood shavings. It does not have any adverse effect on the growth performance, improves feeding behavior, has good effects on foot pad, hock health, and moderate feather condition of Muscovy ducks. From the hygienic view, sand is the best litter to be used as it has the lowest moisture content, water holding capacity, and microbial loads. However, the absence of sex differentiation is a limitation of the current study and further research is needed to determine the effects of sex on ducks reared under different litter types.

## Data Availability

The data presented in this study are available within the article.
